# Harm perception among Swedish daily smokers regarding nicotine, NRT-products and Swedish Snus

**DOI:** 10.1186/1617-9625-8-9

**Published:** 2010-08-13

**Authors:** Tom Wikmans, Lars Ramström

**Affiliations:** 1 Research Group for Societal and Information Studies (FSI), Ingemarsgatan 4B, Stockholm, Sweden; 2 Institute for Tobacco Studies (ITS), Ingemarsgatan 4B, Stockholm, Sweden

## Abstract

**Background:**

In Sweden NRT-products and Snus, are easily available and used as smoking cessation aids. However, most quit attempts are made without any cessation aids. The limited use of these products as cessation aids may be influenced by the way smokers perceive the harmfulness of NRT-products and Snus compared to smoking. The present study examines these perceptions and their association with perceptions of the harmfulness of nicotine itself.

**Methods:**

The study is based on the Swedish part of a two-nation web-based survey of daily smokers in Sweden (n = 1016) and Norway (n = 1000). Questionnaire items addressed perceptions of NRT-products' and Snus' harmfulness and nicotine's part of the health risks of smoking. Data analyses included cross-tabulations and logistic regressions.

**Results:**

A majority, 59% of the answers to the question about harmfulness of NRT-products, and 75% of the answers about harmfulness of Snus, were inconsistent with the scientific evidence by demonstrating exaggerated perceptions of harmfulness. The strongest predictor of consistent answers was the perception of the harmfulness of nicotine. There were also significant associations with own experience of successful use of the products in question. Overall the perceptions of the harmfulness of nicotine were considerably exaggerated. This pattern was more pronounced among women than men. Prevailing misperceptions may be related to the way that different tobacco and nicotine products are presented in the media and other publicly available information sources.

**Conclusions:**

Public information about smoking and health should be expanded to include objective and unambiguous information regarding nicotine's part in the harmfulness of smoking and the harmfulness of different nicotine-containing products compared to smoking.

This is essential in order to preclude that misperceptions regarding these matters could discourage smokers from adopting effective cessation practices with use of nicotine-containing aids.

## Background

### Smoking cessation practices in Sweden

In the late 1970s the prevalence of daily smoking in adult men in Sweden was about 40% and about 35% in adult women [1]. In 2008 the prevalence had come down to 11% among men and 14% among women [2]. This development reflects an expansion of smoking cessation practices that has been influenced by several factors, for example the invention and pioneer use in Sweden of products for nicotine replacement therapy (NRT-products) and the use, mainly among men, of the Swedish kind of oral smokeless tobacco, "Snus", a moist powder of finely ground tobacco leaves with low levels of tobacco-specific nitrosamines. Male prevalence of daily Snus use increased from around 10% to around 22% in the period from the late 1970s to the early 2000s [1]. NRT-products were initially prescription-only medications but already in the late 1980s they became available over the counter in licensed pharmacies and from March 2008 also in many other outlets like grocery stores. Snus has always been sold in a large number of outlets.

The use of the products in question, both NRT-products and Snus, as aids in smoking cessation has been shown to increase the successfulness of quit attempts [3-7]. All these products are easily available in Sweden and they have been used as aids in many quit attempts. Still, the majority of quit attempts in Sweden have been made without use of any cessation aids [7,8]. The limited level of use of cessation aids could be influenced by a number of conditions. One possible such condition could be the way smokers perceive the harmfulness of using NRT-products and Snus compared to smoking.

The purpose of this study is to examine both how daily smokers in Sweden perceive the harmfulness of using nicotine-containing products such as nicotine gum/patch and Swedish Snus, and how they perceive the harmfulness of nicotine itself. Further, the study aims at examining how the harm perceptions of the use of nicotine-containing products are associated with the perception of nicotine's harmfulness and other factors.

## Methods

The current study, VAKT (Habits, Attitudes and Knowledge about Tobacco), is based on the baseline survey of the Swedish part of a prospective panel study of 1016 daily smokers in Sweden and 1000 daily smokers in Norway, that was started in 2009 by the Research Group for Societal and Information Studies (FSI) in cooperation with the Norwegian institute for alcohol and drug research (SIRUS) and Institute for Tobacco studies (ITS).

The study has been approved by the Regional Ethics Review Board in Stockholm, Dnr 2008/2003-31/5.

### Sample selection and data collection

The study population consists of daily smokers born in the period 1950 to 1989 (n = 1016). The participants were recruited from Norstat Inc's web population [9]. In order to avoid selection bias in the sampling process the participants were initially kept unaware that they were recruited to a survey which focused on tobacco use. Those who were identified as daily smokers were asked to participate in a prospective study of their tobacco behaviour. Data collection was done by a web based survey and the participants received approximately 10 SEK ($ 1.30) in compensation for completing the questionnaire.

In order to ascertain the representativity of the sample it was compared with the nationwide representative database Your Country and Your Life (YCYL) with regard to the proportion of men and women, education levels and birth decades in daily smokers of the age groups concerned. Some disparity was observed for the proportion between men and women and for birth decades and the sample was weighted for sex and birth decade. Table 1 shows frequency distributions for the weighted study population, VAKT, and the reference population, YCYL.

**Table 1:** Frequency distributions in the VAKT- and the YCYL-population (in brackets).

Sex	VAKT	YCYL
Women	55.5%	(54.3%)
Men	44.5%	(45.7%)
		
**Education level**	**VAKT**	**YCYL**

Low	72.0%	(78.2%)
High	28.0%	(21.8%)
		
**Birth decades**	**VAKT**	**YCYL**

50s	34.0%	(34.1%)
60s	24.5%	(24.4%)
70s	16.5%	(16.5%)
80s	25.0%	(25.0%)

The questionnaire included questions constructed so that the answers reflected the perception of harmfulness of two kinds of nicotine-containing products (nicotine gum/patch and Swedish Snus) and of nicotine itself. Further there were questions aimed at studying a number of such personal and environmental factors that can be assumed to influence the harm perceptions.

Each one of the questions regarding nicotine-containing products connected to a statement of opinion on which conflicting views had been expressed in different public information channels. The question that was asked about each statement was:

*Can you determine what is true and false? *The wordings of the statements were:

"Long term use of nicotine from patches or gums is almost as harmful to health as smoking"

"Snus use is almost as harmful to the health as smoking"

In the continued text, the question about the first statement is, for short, referred to as the *NRT-question*, the second as the *Snus-question*.

The questions were answered by indicating "True" or "False" or "Do not know". Based on the consensus of scientific opinion illustrated by various studies and reports [10-16] we concluded that for both questions the answer "False" is consistent with the scientific evidence. For the purpose of data analysis the answers were categorized according to whether they represented presence or absence of an outspoken perception consistent with the scientific evidence. Therefore, answer "False" is hereafter referred to as *Consistent*, while answer "True" or "Don't know" is referred to as *Not consistent*.

The question regarding the harmfulness of nicotine was put in the context of how large part of cigarette harm that should be attributed to the nicotine in the smoke. In the continued text, this question is, for short, referred to as the *Nicotine-question*. The wording was:

"According to you, how large part of the health risks in smoking comes from the nicotine?"

The answers were coded into four categories: "None or very small part", "Relatively small part", "Relatively large part", "Very large part or all". According to the consensus of scientific opinion expressed in, for example, some of the sources mentioned above, the first two answers are more correct than the two last ones.

### Data analysis

Descriptive analyses by, cross-tabulations were performed to examine the distribution of frequencies for different answers among men and women.

Logistic regressions using SPSS 13.0 were performed to calculate the odds ratios for consistent answers to the *NRT-question *and the *Snus-question *controlling for "Perception of nicotine's part of the health risks in smoking", "Birth decade", "Education level", "Sex", "Desire to stop smoking", "Place of residence", "Time to first cigarette", "Previous use of NRT and benefits thereof in attempts to quit smoking", "Previous use of Snus and benefits thereof in attempts to quit smoking", "Proximity to people that have used NRT in attempts to quit smoking" and "Proximity to people that have used Snus in attempts to quit smoking".

## Results

### Descriptive analyses

Both in men and women a majority of the respondents answered *Not consistent *on both the *NRT-question *and the *Snus-question*. Both questions yielded larger proportion of consistent answers from men than from women. For details, see Table 2.

**Table 2:** Answers to the NRT-question and Snus-question for women and men

	NRT-question			Snus-question		
	**Sex**			**Sex**		
	**Women**	**Men**	**Total**	**Women**	**Men**	**Total**

Not consistent	62.1%	55.3%	59.1%	81.6%	65.8%	74.5%
Consistent	37.9%	44.7%	40.9%	18.4%	34.2%	25.5%
n =	549	445	994	549	445	994
Missing values	15	7	22	15	7	22

Among women 37.3% answered "Don't know" (subcategory of *Not consistent *answers) to the *NRT-question*, while the corresponding proportion of men is 40.2%. For the *Snus-question *the proportion answering "Don't know" is 12.0% for women and 20.7% for men.

The answers to the *Nicotine-question *demonstrate considerably exaggerated perceptions of the harmfulness of nicotine. This pattern is more pronounced among women than among men. For details, see Table 3.

**Table 3:** Answers to the Nicotine-question for women and men

Nicotine's part of the health risks of smoking	Sex		
	**Women**	**Men**	**Total**

None or very small part	8.5%	22.1%	14.6%
Relatively small part	26.2%	36.3%	30.7%
Relatively large part	35.6%	25.2%	31.0%
Very large part or all	29.6%	16.4%	23.7%
n =	550	444	994
Missing values	15	7	22

### Regression analyses

The results from the regression analyses are presented in Table 4.

**Table 4:** Logistic regressions predicting assessments consistent with scientific evidence regarding statements on NRT and Snus.

	Long term use of nicotine from patches or gums is almost as harmful to **health as smoking**.			Snus use is almost as harmful to the health **as smoking**.		
		
Predictors	p-value	OR	**95% C.I**.	p-value	OR	**95% C.I**.
**Perception of nicotine's part of the health risks in smoking**						
None or very small part (ref)		1			1	
Relatively small part	0.01	0.48	0.29-0.80	0.00	0.44	0.26-0.75
Relatively large part	0.00	0.20	0.12-0.35	0.00	0.30	0.17-0.54
Very large part or all	0.00	0.25	0.14-0.43	0.00	0.33	0.18-0.61
**Birth decade**						
80 s (ref)		1			1	
70s	0.17	0.70	0.43-1.16	0.29	0.75	0.44-1.28
60s	0.97	0.99	0.63-1.56	0.52	0.85	0.52-1.39
50s	0.46	1.19	0.76-1.86	0.10	0.65	0.39-1.08
**Education level**						
Low (ref)		1			1	
High	0.00	1.88	1.34-2.64	0.19	1.30	0.88-1.93
**Sex**						
Women (ref)		1			1	
Men	0.22	1.23	0.89-1.70	0.05	1.43	0.10-2.07
**Desire to quit smoking**						
No desire (ref)		1			1	
Some desire	0.80	0.92	0.47-1.80	0.60	1.23	0.57-2.65
Strong desire	0.93	1.03	0.51-2.09	0.80	0.90	0.40-2.03
**Place of residence**						
Rural areas (ref)		1			1	
Small city 4'-30'	0.16	1.37	0.88-2.13	0.31	0.76	0.45-1.29
Large city >30'	0.90	1.03	0.66-1.62	0.55	1.17	0.70-1.96
Main city	0.03	1.65	1.05-2.57	0.47	1.21	0.72-2.02
**Time to first cigarette (after waking up)**						
>30 minutes (ref)		1			1	
6-30 minutes	0.68	1.07	0.77-1.50	0.03	1.57	1.05-2.35
> 5 minutes	0.76	1.07	0.68-1.70	0.01	2.03	1.20-3.42
**Previous use of NRT and benefits thereof in attempts to quit smoking**						
Not used (ref)		1			1	
Used, none or modest benefit	0.70	1.08	0.74-1.55	0.02	0.61	0.40-0.94
Used, quite a large benefit or helped completely	0.01	1.71	1.14-2.57	0.17	0.72	0.45-1.15
**Previous use of Snus and benefits thereof in attempts to quit smoking**						
Not used (ref)		1			1	
Used, none or modest benefit	0.37	0.81	0.51-1.28	0.00	3.11	1.92-5.04
Used, quite a large benefit or helped completely	0.95	1.01	0.67-1.53	0.00	3.88	2.50-6.01
**Proximity to people that have used NRT in attempts to quit smoking**						
No proximity (ref)		1			1	
Proximity to unsuccessful attempts	0.45	1.17	0.78-1.76	0.68	1.10	0.69-1.77
Proximity to successful attempts	0.76	1.07	0.70-1.63	0.74	1.09	0.66-1.79
**Proximity to people that have used Snus in attempts to quit smoking**						
No proximity (ref)		1				
Proximity to unsuccessful attempts	0.37	1.22	0.79-1.87	0.38	0.80	0.48-1.33
Proximity to successful attempts	0.01	1.60	1.11-2.32	0.39	1.20	0.79-1.85

The answers to the *NRT-question *were most strongly influenced by the perception of nicotine's part of the health risks in smoking. Among those who (correctly) perceived nicotine's harmfulness as low, the likelihood of *Consistent *answer to the *NRT-question *was about four times as high as among those who perceived nicotine's harmfulness as high. Among those with high education level the likelihood of a *Consistent *answer to the *NRT-question *was almost double as high as among those with low education. Similarly, residence in a main city was associated with higher likelihood of *Consistent *answer than residence in rural areas. Successful previous use of NRT in attempts to quit smoking, as well as proximity to people that had successfully used Snus in attempts to quit smoking, also had an association with *Consistent *answers to the *NRT-question*.

The answers to the *Snus-question *were also most strongly influenced by the perception of nicotine's part of the health risks in smoking. Among those who perceived nicotine's harmfulness as low, the likelihood of *Consistent *answer to the *Snus-question *was about three times as high as among those who perceived nicotine's harmfulness as high. Birth in the 1980s was associated with slightly higher likelihood of C*onsistent *answer than birth in the 1950s, and men were more likely than women to give *Consistent *answers. Highest level of nicotine dependence (< 5 minutes to first cigarette) was associated with higher likelihood of *Consistent *answer than the lowest level (> 30 minutes). Previous use of Snus in attempts to quit smoking also had a strong association with *Consistent *answers to the *Snus-question*.

The predictors "Birth decades" and "Desire to stop smoking" had no significant association with either the *NRT-question *or the *Snus-question*. Men and women do not differ significantly with regard to the *NRT-question*, while there is a borderline significance (at the 95% level) for higher likelihood of Consistent answers to the *Snus-question *from men than from women. With regard to the *NRT-question*, residents in main cities give *Consistent *answers more often than residents in rural areas. With regard to the *Snus-question *there are no differences between residence areas. Other predictors included in the model did not show significant associations.

## Discussion

In this study we have asked questions to examine how daily smokers in Sweden perceive the harmfulness of using nicotine-containing products and how they perceive the harmfulness of nicotine itself. The answers could be based either on correct or on false information or on own experiences and/or environmental influences of different kinds. Therefore the answers will in most cases reflect "believed knowledge" rather than cognizance of established evidence. But believed knowledge can have, and in many cases has, equal consequences as real knowledge, so as pointed out by W.I. Thomas in the Thomas theorem: "If men define situations as real, they are real in their consequences." [17].

Overall, the findings reveal that Swedish smokers have exaggerated perceptions of the harmfulness of nicotine and nicotine-containing products. Similar findings have also been reported in American studies. A study of college freshmen [18] found that around 20% of the respondents did erroneously perceive nicotine gum/patch to be as harmful as or more harmful than cigarettes and almost 90% of them did erroneously perceive smokeless tobacco products in the same way. Another American study [19] highlighted the potential role of exaggerated perceptions of the health risks of nicotine, the key substance in all these products. For example did around two thirds of the respondents erroneously believe that nicotine causes cancer.

In the descriptive analysis we examined to what extent the different harm perceptions were consistent with the scientific evidence among men and women. We found that a majority of Swedish daily smokers have a believed knowledge, both about NRT-use and about Snus-use harmfulness compared to smoking that is not consistent with scientific evidence. Absence of correct believed knowledge is more common for Snus use than for NRT use. One possible interpretation could be that NRT-products have gained some confidence by their status as medicinal products. In both cases the absence of correct believed knowledge is more common among women then among men, the difference being larger for Snus use than for NRT use. With regard to perceptions of the harmfulness of nicotine itself the study does again demonstrate a frequent absence of correct believed knowledge. More than half, 54%, of the answers are on the lesser correct half of the scale. Again, the absence of correct believed knowledge is more common among women then among men.

In the regression analyses we examined how the harm perceptions of NRT-use and Snus use were associated with a number of possible predictors. Among those predictors include in the regression model strong influences were found for the perception of nicotine harm and Own experience of successful use of the products in question, while most others had weak or no association (for details, see the Results section).

A person's perceptions of a product or a substance can be seen as the outcome of an accumulated intake of information and impressions from various sources. According to the theories of Selective Exposure and Selective Perception [20-22] people will tend to choose which information to take note of and to adopt interpretations that are preferable with respect to personal conditions. This can, for example, make people who are users of a certain product more likely to develop a positive perception of that product than non-users. This kind of mechanisms may be part of the reason behind the finding that men, a group with many Snus users, have slightly higher ORs for consistent answers to the *Snus-question *than women, a group with few Snus users. The influence of personal conditions is further illustrated by the findings that people with own experience of successful NRT use for smoking cessation have higher ORs than non-users for consistent answers to the *NRT-question*, just as all ORs for consistent answers to the *Snus-question *are higher among those with own experience of Snus use for smoking cessation. Further, an exaggerated perception of nicotine harm may, particularly among people without own experience of use of nicotine products, increase the likelihood of selectivity in choosing and interpreting various impressions in such a way that supports a perception of nicotine products being *a priori *harmful.

The outcome of a person's accumulated intake of information will evidently first of all depend on what information is available and understandable altogether. In Swedish media there has been very modest coverage of different NRT-products. Messages regarding Snus often have a one-sided nature with main focus either on pros or cons. A striking example of contradictory messages was presented on 10^th ^of May 2007 in the two major Swedish morning newspapers. Both reported an article from The Lancet, one by saying: "Twice the risk for Snus users to get cancer" [23] and the other by saying "Snus can be beneficial for smokers" [24]. Overall, the pictures about different tobacco and nicotine products presented in the media, as well as in other publicly available information sources, are not concordant and can be both confusing and misleading for the receiver. We found that high education increases the likelihood of consistent answers to the *NRT-question*. One possible interpretation of this could be that education constructs a ground that makes it easier to assess relevant information about NRT-products. The finding that high education does not influence the likelihood of consistent answers to the *Snus-question *is consistent with findings in an American study showing that university staff members had no better knowledge than others with regard to smokeless tobacco [25]. This might reflect the lack of unambiguous information about Snus. This interpretation is further supported by the overall picture presented in Table 2 showing that consistent answers are less frequent for the *Snus-question *than for the *NRT-*question.

It appears that public information channels in Sweden have failed to effectively communicate pertinent pieces of correct information on nicotine and nicotine products. The findings from this study on nicotine and NRT-products are consistent with those of American studies [18,19], and analogous patterns are found for Swedish Snus as well. It can be assumed that similar patterns are prevailing in most countries. While further research on these matters will be needed, the current, widespread occurrence of false believed knowledge must in any case be seen as an unfortunate situation. Perceptions of these kinds are likely to influence whether smokers choose to use a nicotine-containing aid to try to quit smoking, or choose to continue to smoke. As pointed out in a recent study of different scenarios [26] these choices are critical to the attainment of the goals of reducing smoking prevalence. It will therefore be essential to expand public, non-commercial information on smoking and health to include comprehensive data regarding the part nicotine plays in the harmfulness of smoking as well as objective data on the harmfulness of different nicotine-containing products compared to smoking.

## Conclusions

Overall there is a high degree of believed knowledge regarding NRT-products and Snus that is not consistent with scientific evidence and it is most strongly influenced by the misbelief that nicotine plays a large part in the harmfulness from smoking. It is therefore needed to expand public, non-commercial information on smoking and health to include comprehensive data regarding the part nicotine plays in the harmfulness of smoking as well as objective and unambiguous data on the harmfulness of different nicotine-containing products compared to smoking. This is essential in order to preclude that misperceptions regarding these matters could discourage smokers from adopting effective cessation practices with use of nicotine-containing aids.

## Competing interests

TW has no competing interest.

LR has no competing interest. Owns shares in Pfizer Inc.

## Authors' contributions

TW conceived the research idea and held the primary responsibility for study design and data collection. LR evaluated the literature and participated in finalizing the study design. TW and LR participated in analysis and interpretation of data and writing of the report. Both authors read and approved the final manuscript.
